# A Superamphiphobic Sponge with Mechanical Durability and a Self-Cleaning Effect

**DOI:** 10.1038/srep29993

**Published:** 2016-07-20

**Authors:** Daewon Kim, Hwon Im, Moo Jin Kwak, Eunkyoung Byun, Sung Gap Im, Yang-Kyu Choi

**Affiliations:** 1 School of Electrical Engineering, Korea Advanced Institute of Science and Technology (KAIST), 291 Daehak-ro, Yuseong-gu, Daejeon 34141, Republic of Korea; 2 Department of Chemical & Biomolecular Engineering, Korea Advanced Institute of Science and Technology (KAIST), 291 Daehak-ro, Yuseong-gu, Daejeon 34141, Republic of Korea; 3 IT&E Materials R&D, LG Chem Research Park, 188 Munji-ro, Yuseong-gu, Daejeon 34122, Republic of Korea

## Abstract

A robust superamphiphobic sponge (SA-sponge) is proposed by using a single initiated chemical vapor deposition (i-CVD) process. Poly(3,3,4,4,5,5,6,6,7,7,8,8,9,9,10,10,10-heptadecafluorodecyl methacrylate) (PFDMA) is deposited on a commercial sponge by the polymerization of fluoroalkyl acrylates during the i-CVD process. This PFDMA is conformally coated onto both the exterior and interior of the sponge structure by a single step of the i-CVD process at nearly room temperature. Due to the inherent porous structure of the sponge and the hydrophobic property of the fluorine-based PFDMA, the demonstrated SA-sponge shows not only superhydrophobicity but also superoleophobicity. Furthermore, the fabricated SA-sponge is robust with regard to physical and chemical damage. The fabricated SA-sponge can be utilized for multi-purpose applications such as gas-permeable liquid separators.

Wettability is one of the most important characteristics of the surface of a solid material. Many solid surfaces in nature, such as the lotus leaf and cicada wing, exhibit notable superhydrophobicity^1^,^2^. Such superhydrophobicity is quantitatively characterized by a water contact angle greater than 150° and a sliding angle of less than 10°^ ^3,4,5,6. Many researchers have intensively investigated superhydrophobicity using various methods, such as chemical etching^7^, layer-by-layer deposition^8^, colloidal coating^9^, hydrothermal synthesis^10^, electro-spinning^11^, and anodic oxidation^12^ for various applications of superhydrophobic surfaces in both pure science and engineering fields^13^,^14^. On the other hand, superoleophobicity, referring to the capability to repel oil, has a wider range of multi-purpose applications compared to superhydrophobicity. These include self-cleaning, anti-fouling, anti-icing, anti-corrosion, and drag reduction applications^15^,^16^,^17^,^18^. In general, superoleophobic surfaces show superhydrophobic properties as well because a superoleophobic material has lower surface tension for both water and oil as compared to a superhydrophobic surface^19^,^20^. Superamphiphobicity refers to the capability to repel both water and oil. Thus, it has attracted much attention recently due to its strong potential for applications which demand hydrophobicity, oleophobicity, anti-pollution capabilities, a self-cleaning ability, and low friction^21^,^22^. Further, it is easily inferred that the fabrication of superamphiphobic surface which repels both water and oily liquids is typically difficult to realize.

Generally, the chemical composition and the geometrical structure of a surface are the key factors to achieve low surface energy of an amphiphobic substrate. Several complicated attempts to create a superamphiphobic surface have been reported^19^,^20^,^23^,^24^,^25^,^26^,^27^. However, such attempts are not only time-consuming owing to the multiple fabrication steps but also require a vacuum process or a relatively high temperature process providing a limitation to the selection of the target substrate. Another limiting factor is the very small number of available substrates.

A porous sponge based on polyurethane (PU) is an example of a commercially available 3D porous material^28^,^29^. A commercially available PU sponge can harness a superamphiphobic property due to its inherent pore structure, and its intrinsic excellent elasticity is attractive for the durability. Especially, due to its very high surface-to-volume ratio, many studies have been reported that a porous sponge is broadly utilized for catalyst, lithium ion-battery, and cell growth templates^30^,^31^,^32^. However, they are also typically hydrophilic. Therefore, modifications are essential for conversion from a hydrophilic to an oleophobic state.

The initiated chemical vapor deposition (i-CVD) process used as part of our fabrication method is an emerging one-step process with good versatility^33^,^34^,^35^,^36^,^37^. It does not cause contamination or damage by solvents, and it allows a conformal coating of which the thickness is controllable. It should be noted that the step coverage is nearly perfect to wrap even an irregular shaped pore and skeleton, which are in deeper inside of the sponge than 1 cm. This step coverage has not been reported ever. Furthermore, the i-CVD process is an all-dry polymerization process. And such polymerization can occur near room temperature ranging from 15 to 40 °C. Further, a thin layer deposition is available for most solid materials. A vaporized monomer and an initiator are used for radical polymerization in the vapor phase, and the radicals are formed by vaporized initiator molecules that are thermally decomposed through contact with hot filaments. The radicals initiate a chain reaction with the vaporized monomers at nearly room temperature, and the polymers are deposited onto the substrate conformally regardless of the substrate material. Different from liquid-phase polymerizations, the i-CVD process consists of synthesizing the polymer on the surface of a desired substrate as well as vaporizing the monomer and the initiator in a chamber. Deposition of polymers with vapor phase is advantageous in many aspects compared to conventional liquid-phase deposition^38^. One major advantage of the vapor deposition of polymers is that the substrate onto which the polymers are deposited, is not damaged by the deposition process. In the case of the liquid-phase deposition, the exposure to the solvents used in the polymerization often causes damage to the substrate that is vulnerable to liquids. Different from the conventional CVD, the i-CVD process is available to deposit the polymer on nearly all the solid-phase materials due to mild deposition condition. In the conventional CVD, it is possible to deposit only inorganic materials because of high temperature above 500 °C, which can deform the organic materials^39^.

Here, in this work, we demonstrate a superamphiphobic sponge which is robust to physical and chemical damage *via* the simple one-step i-CVD process, which is an all-dry vapor-phase process that does not require any solvents. Moreover, the i-CVD process takes place at room temperature, which is attractive for various vulnerable substrates such as fabrics and papers that are prone to thermal damage. On the other hand, solution-based coating methods can be possibly damage the substrate due to the diffusion of functionalizing solutions into the bulk of the porous substrate arising from the capillary effect. Further, superior mechanical durability and high chemical stability of the fabricated superamphiphobic sponge were clearly verified through the physical and chemical test. This superamphiphobic sponge *via* i-CVD process can be potentially a promising candidates for commercial products, such as a gas-permeable liquid separator.

## Results and Discussion

### Superamphiphobic sponge by i-CVD

**Figure 1:**
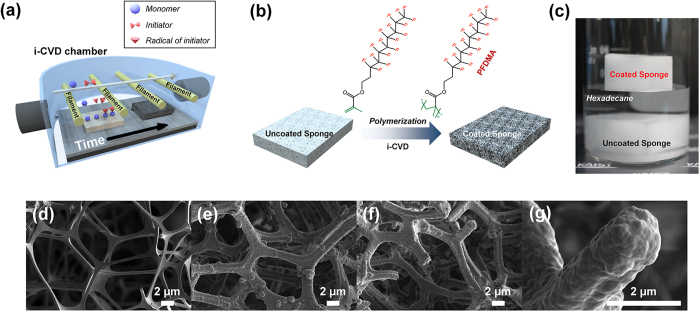
(**a**) Schematic illustration of the i-CVD process for SA-sponge fabrication. (**b**) Reaction scheme for polymerization of fluoro-alkyl acrylates by the i-CVD process. (**c**) Digital camera snapshot of the SA-sponge and bare sponge submerged in hexadecane solvent. Morphologies of (**d**) skeleton in bare sponge, (**e**) skeleton in outside of the SA-sponge, (**f**) skeleton in inside of the SA-sponge, and (**g**) skeleton of the SA-sponge at high resolution using SEM. All scale bars are 2 μm.

Figure 1a shows a schematic illustration of the fabrication procedure used to create the superamphiphobic sponge (SA-sponge) by the i-CVD process. The i-CVD process is an all-dry vapor-phase technique in which vaporized monomers form polymeric films directly on the surface of a substrate. Inherited from the conventional CVD processes, the i-CVD process harnesses the good scalability and compatibility with high-throughput manufacturing^40^,^41^. In addition, it can be set up mostly with a standard protocol. Polymeric films in the i-CVD are formed by the following mechanism: (i) introduction of vaporized monomers and initiators; (ii) thermal dissociation of initiators to form radicals upon contact with heated filaments; (iii) physical adsorption of monomers onto cooled substrates; and (iv) free-radical polymerization of adsorbed monomers on the surface. The surface-growing characteristics of the i-CVD process enable the conformal step coverage of the polymeric film with a high degree of uniformity over a large area with virtually no surface or substrate limitations^42^. Moreover, the polymer film deposited by i-CVD generally has high chemical purity with no residue because the deposition process is inherently free of solvents and additives.

The fabrication scheme of the SA-sponge is shown in Fig. 1b. The polyurethane-based highly porous sponge was a commercially available sponge source from a nearby market. A piece of the hydrophilic sponge was conformally coated with a 100-nm-thick hydrophobic polymer film layer of poly(3,3,4,4,5,5,6,6,7,7,8,8,9,9,10,10,10-heptadecafluorodecyl methacrylate) (PFDMA) *via* the i-CVD process^43^,^44^,^45^. In general, polymers containing long fluoro-alkyl chains such as PFDMA are known to exhibit a high degree of water repellency, with the water contact angle exceeding 150° on rough surfaces, as well as extremely low contact-angle hysteresis. The i-CVD process provided a conformal PFDMA coating on all skeletons of the exterior and interior sponge with the one-pot i-CVD coating step. As previously reported, the vapor-phase polymerization process allowed the infiltration of vaporized initiators and monomers through the pores in the sponge^46^. For the direct verification of the successful fabrication of the sponges, two types of sponges, which include a PFDMA-coated sponge and a bare sponge, were immersed in a vial filled with hexadecane, as shown in Fig. 1c. It was clearly found that the SA-sponge floated on the hexadecane, whereas the uncoated sponge sunk in the hexadecane, as it absorbed this solvent. Further, the PFDMA film conformally coated by the i-CVD process was optically transparent hence there is no change in color between the PFDMA coated and uncoated (bare) sponge.

### Characterization and analysis

To confirm the conformal coating on the skeleton of the sponge from outside to inside, the morphology of the sponge was investigated before and after the i-CVD deposition process by scanning electron microscopy (SEM), as shown in Fig. 1d to 1g. A thickness of the bare sponge skeleton and its pore size are approximately 1 μm and 20 μm, respectively as shown in Fig. 1d. Figure 1e shows the exterior skeleton of the SA-sponge. This figure clearly shows that the polymer was conformally coated onto the skeleton of the sponge. Additional SEM images for SA-sponge are shown in Figure S1. Given that vaporized initiators and monomers can infiltrate into the pores, the interior skeleton of the SA-sponge was also conformally coated with the PFDMA and the thickness of the PFDMA in the interior is identical to that of the exterior, as shown in Fig. 1f.

**Figure 2:**
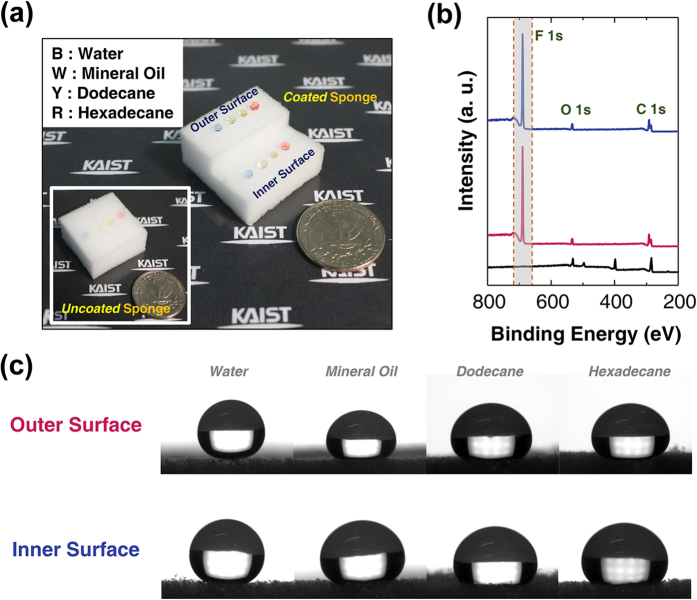
(**a**) Snapshot of various liquid droplets (water: blue-B, mineral oil: white-W, dodecane: yellow-Y, hexadecane: red-R) on the SA-sponge. The inset image shows the same liquid droplets on the bare sponge. (**b**) X-ray photoelectron spectroscopy (XPS) shows each spectrum of the bare sponge (black), outside surface of the SA-sponge (red), and inside surface of the SA-sponge (blue). (**c**) Photographs of contact angles of aforementioned liquid droplets on the inside and outside surface of the SA-sponge. All the contact angles exceed 150°.

Various solvents: water (blue, B) (γ = 72.8 mN/m), mineral oil (white, W) (γ = 47.26 mN/m), dodecane (yellow, Y) (γ = 25.35 mN/m), and hexadecane (red, R) (γ = 27.47 mN/m) were prepared to measure the static contact angle of the SA-sponge, as shown in Fig. 2a. Here, γ is the surface tension obtained from the manufacturer of each solvent, which represents the cohesive force between liquid molecules. To confirm as well that the PFDMA was coated onto the inside surface of the SA-sponge, the fabricated SA-sponge was cut into two regions. To check the chemical composition of the SA-sponge surfaces, a XPS spectrum analysis was conducted on the outside and inside surfaces of the SA-sponge. As shown in Fig. 2b, the peak of F 1s was drastically increased on the inside and outside surface of the sponge compared with the uncoated bare sponge. No peak of fluorine implies that any fluorine-containing group is not existed in the bare sponge, while each high peak of the F 1s means that the PFDMA is coated even in the inside as well as in the outside of the sponge. And it is noteworthy that two peaks of F 1s are nearly identical in the inside and outside of the sponge, which conveys that the PFDMA was notably coated in the inside and outside of the sponge skeleton. To further analyze the surface of SA-sponge, fourier transform infrared (FTIR) spectrums of the PFDMA polymer bare sponge (black), inside surface of PFDMA coated sponge, (red), and outside surface of PFDMA coated sponge (blue) were obtained, as shown in Figure S2. The intensity for the −CF3 peak in after the deposition of PFDMA by use of the i-CVD drastically increased compared to that of the bare sponge, a control sample.

The static contact angle measurements of inside and outside the surface of the SA-sponge demonstrated that all surfaces coated with PFDMA by the i-CVD process exhibited superamphiphobicity, as follows: 158 ± 4° (water), 151 ± 4° (mineral oil), 150 ± 5° (dodecane), and 149 ± 6° (hexadecane), as shown in Fig. 2c. In addition, with regard to the SA-sponge, they showed identical static contact angles on the inside and outside surfaces. These findings verify that the skeleton of the sponge was conformally coated with the PFDMA, not only on the outer surface but also on the inner surface *via* the i-CVD process. Such a uniform coating of the outer and inner surfaces of the SA-sponge guarantee both superhydrophobicity and superoleophobicity throughout the structure of the sponge.

### Durability for physical stress

**Figure 3:**
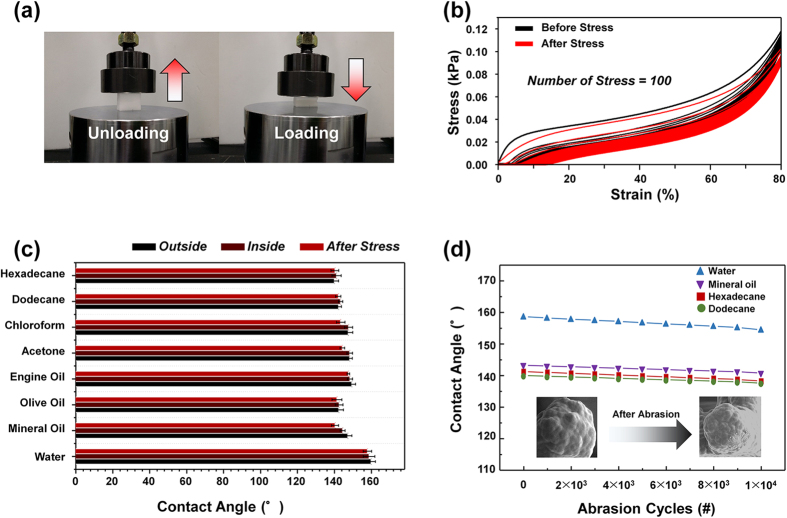
Physical durability test and static contact angle of SA-sponge. (**a**) Digital snapshot of experimental setup for stress-strain characteristics using a universal testing machine (UTM) with (left) unloading state and (right) loading state. (**b**) Stress-strain curve of SA-sponge (black) before the test and (red) after test using UTM. (**c**) Static contact angle of various liquids before/after stress tests. (**d**) Static contact angle for four liquids (water, mineral oil, dodecane, hexadecane) after abrasion test for 10000 cycles.

To estimate the durability of the fabricated SA-sponge under physical stress, iterative compress tests (100 times) of the SA-sponge and a bare sponge under various strain ranges from 0 to 80% were performed, as shown in Fig. 3a. The stress-strain curve in Fig. 3b shows that the elastic modulus of the PFDMA-coated SA-sponge was lower than that of the bare sponge. Due to the fact that all skeletons of the sponge were coated with the PFDMA, the elastic modulus of the SA-sponge is slightly decreased. Although external physical stress was repeatedly applied to the SA-sponge, the static contact angles of the various liquid droplets did not change, as shown in Fig. 3c. This arises from two factors. First, many pores with skeletons in the SA-sponge were reversibly recovered, and second, the PFDMA layer was not damaged, thus supporting the strong adhesion between the skeleton of the sponge and the polymer as the iterative external stress was applied. Further, it is seen that the values of contact angle of outside surface exhibit more slightly larger than those of inside surface. This tendency is closely correlated with a thickness of PFDMA to enclose fluorine-containing polymer. As a depth of the sponge is increased, the thickness of PFDMA is gradually decreased due to the fact that the distance between the i-CVD chamber and target surface is increased thereby a growth rate is decreased.

**Figure 4:**
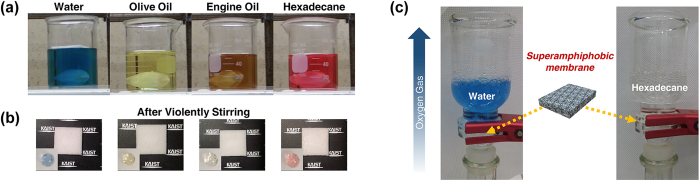
(**a**) Digital snapshots to show chemical immunity of the SA-sponge. The SA-sponge is immersed in four liquids (water: blue, olive oil: yellow, engine oil: brown, hexadecane: red) with magnetic bar for vigorous stirring. (**b**) Photographs of the SA-sponge taken out after being submerged in the above liquids and each droplet on surface of the SA-sponge shown in inset image. (**c**) Digital camera images of gas permeable liquid separator (water: left, dodecane: right) using the fabricated SA-sponge.

For an alternative confirmation of the durability against external mechanical stress, an additional abrasion test was performed on the SA-sponge using an electrodynamic shaker. In the previous compress test, a mechanical head, which is always contacted with top surface of the sponge (*i.e.*, the distance between the head and the sponge is zero regardless of a non-squeezed or squeezed state), applies pressure in a range of from 0 Pa to 100 Pa to the sponge *via* iterative squeezing processes. In the abrasion test, the head, which is separated from top surface of the sponge, hits the top surface of the sponge with the pressure of 30 kPa. Thus the top surface of the sponge can be damaged by such striking process. By keep tracing of a change of top surface morphology with the aid of SEM, the surface damage is directly observed. The configuration of this experimental setup is schematically illustrated in Figure S3. Despite the fact that the nanoscale surface protrusions were partially damaged after the 10000 cycles of the abrasion test, the static contact angles of the various liquid droplets scarcely changed as the test cycles continued. This characteristic is ascribed to the PFDMA layer, which remained strongly adhered to the skeleton of the SA-sponge, even after 10000 cycles of the abrasion test in Fig. 3d. The inset images of Fig. 4d indicate that surface morphology of the PFDMA-coated sponge was hardly changed during the abrasion test and confirm that it can endure physical stress up to 30 kPa.

### Stability for chemical damage

Figure 4a shows various types of liquids in which the SA-sponge was submerged in order to investigate the effects of chemical damage. Once the SA-sponge was submerged into the four liquids (water, olive oil, engine oil and hexadecane) the beaker in each case was vigorously stirred for 6 hours using a magnetic bar spinning at a high RPM (revolutions per minute) of 5000. After vigorous stirring, the SA-sponge was taken out of the solution and tapped against a table twice to shake off any liquid residue. The clean surfaces of the SA-sponge after being removed from the various liquids are shown in Fig. 4b. Despite the vigorous stirring, the superamphiphobic property of the SA-sponge was undamaged, confirming the chemical immunity of the SA-sponge given the unchanged contact angles compared to those of the as-fabricated pristine SA-sponge. Figure 4c shows the experimental setup, in which the SA-sponge is used as a gas-permeable liquid separator for water (left) and oil (right). From a tube on the bottom of the beaker, gas is supplied to the beaker. The gas penetrates the SA sponge, while the liquids fail to pass through the SA-sponge. Therefore, the SA-sponge can be utilized as a gas-permeable liquid separator, with applications to commercial products.

## Conclusion

In summary, we demonstrated a superamphiphobic sponge that repels both water and oil *via* the i-CVD process with a porous sponge. With the aid of the i-CVD process, the PFDMA was conformally coated onto the sponge structure with a single i-CVD step at nearly room temperature. Due to the porous nature of the sponge and the inherent hydrophobicity of the fluorine-based PFDMA which was uniformly coated throughout the sponge, the demonstrated SA-sponge shows superhydrophobicity and superoleophobicity. Furthermore, it was verified that the fabricated SA-sponge was robust against physical and chemical damage. The superhydrophobic and superoleophobic SA-sponge fabricated with the simple i-CVD process with high immunity to physical and chemical damage can be utilized for multi-purpose applications. As an example, a gas-permeable liquid separator was demonstrated.

## Methods

### Polymerization onto sponge by i-CVD

Poly(3,3,4,4,5,5,6,6,7,7,8,8,9,9,10,10,10-heptadecafluorodecyl methacrylate) (PFDMA) was deposited onto a commercial sponge (Daiso, Korea) with size of 5 **×** 5 cm2 and a height of 3 cm by use of the i-CVD process. The monomer, 3,3,4,4,5,5,6,6,7,7,8,8,9,9,10,10,10-heptadecafluorodecyl methacrylate (FDMA, 97%), and the initiator, *tert*-butyl peroxide (TBPO, 98%), were purchased from Sigma Aldrich and were used as received. Both the FDMA and the TBPO were loaded into separate source cylinders, which were introduced into the i-CVD reactor (Daeki Hi-Tech, Korea) at a flow rate of 1 cm3/min (sccm) in each case. A 100 nm thin film of PFDMA was conformably deposited onto the substrates at a rate of 17 nm/min. The reaction pressure and substrate temperature were kept at 80 mTorr and 37 °C, respectively.

### Characterization

The morphology of the PFDMA-coated sponge was observed using a scanning electron microscope (SEM) with an operation voltage of 2 kV (Magellan 400, FEI, USA). Fourier transform infrared spectroscopy (FTIR) of the PFDMA-coated sponge before and after the deposition process was performed with a Nicolet iS50 device (Thermo Scientific, USA). X-ray photoelectron spectroscopy (XPS) data were obtained using a K-alpha (Thermo Scientific, USA) with a monochromatized Kα source. The thickness and growth rate of coating were measured by He-Ne laser (JDS Uniphase, USA) interferometer which was attached to the chamber of i-CVD^43^,^46^. The static contact angle was measured by a contact angle analyzer (Phoenix 150, SEO, Korea) equipped with a microsyringe that dispenses a 6 μL water droplet. Mechanical tests were carried out on a universal testing machine (INSTRON 5583, Instron Corporation, USA) at a rate of 0.1 mm/min with a 1000 N load cell. An abrasion durability test was conducted using a electrodynamic shaker (LW140.141-110, Labworks Inc., USA).

## Additional Information

**How to cite this article**: Kim, D. *et al.* A Superamphiphobic Sponge with Mechanical Durability and a Self-Cleaning Effect. *Sci. Rep.* **6**, 29993; doi: 10.1038/srep29993 (2016).

## Supplementary Material

Supplementary Information

Supplementary Video S1
