# Supplementary material for: A Superamphiphobic Sponge with Mechanical Durability and a Self-Cleaning Effect
Source: Sci Rep. 2016 Jul 20;6:29993. doi: 10.1038/srep29993 (PMC4951731; doi:10.1038/srep29993)
Supplement: Supplementary Information [file srep29993-s1.doc]

**Supporting Information**

A Superamphiphobic Sponge with Mechanical Durability and a Self-cleaning Effect

Daewon Kim,^1^ Hwon Im,^1^ Moo Jin Kwak,^2^ Eunkyoung Byun,^3^ Sung Gap Im,^2^* and Yang-Kyu Choi^1^*

^1^ School of Electrical Engineering, Korea Advanced Institute of Science and Technology (KAIST), 291 Daehak-ro, Yuseong-gu, Daejeon 34141, Republic of Korea

^2^ Department of Chemical & Biomolecular Engineering, Korea Advanced Institute of Science and Technology (KAIST), 291 Daehak-ro, Yuseong-gu, Daejeon 34141, Republic of Korea

^3^ IT&E Materials R&D, LG Chem Research Park, 188 Munji-ro, Yuseong-gu, Daejeon 34122, Republic of Korea

* Address correspondence to ykchoi@ee.kaist.ac.kr, sgim@kaist.ac.kr

**Table of contents**

1. **Additional SEM image of PFDMA coating onto sponge**
2. **FTIR spectrum of the PFDMA coated sponge**
3. **Experimental setup for abrasion test**
4. **TEM image for conformal coating by use of the i-CVD**
5. **Thickness of PFDMA thin layer according to depth**
6. **Video for a superamphiphobic sponge in various liquids**

**1. Additional SEM image of PFDMA coating onto sponge**


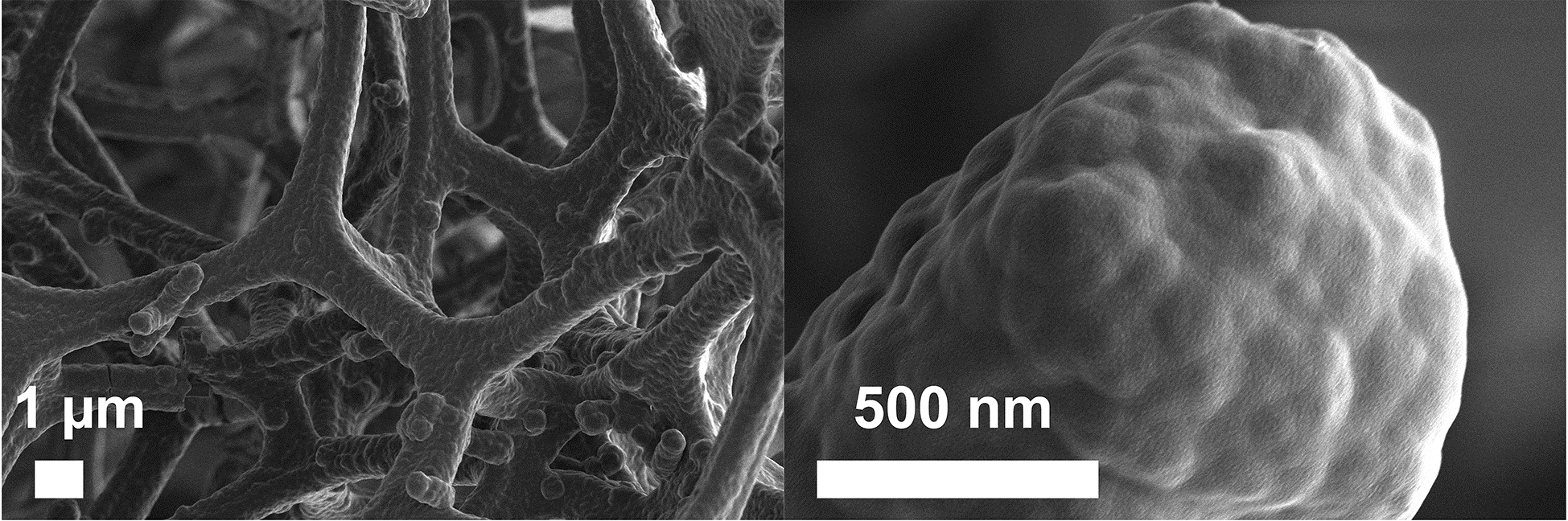


**Figure S1.** Morphologies of PFDMA coated sponge by using SEM.

**2. FTIR spectrum of the PFDMA coated sponge**


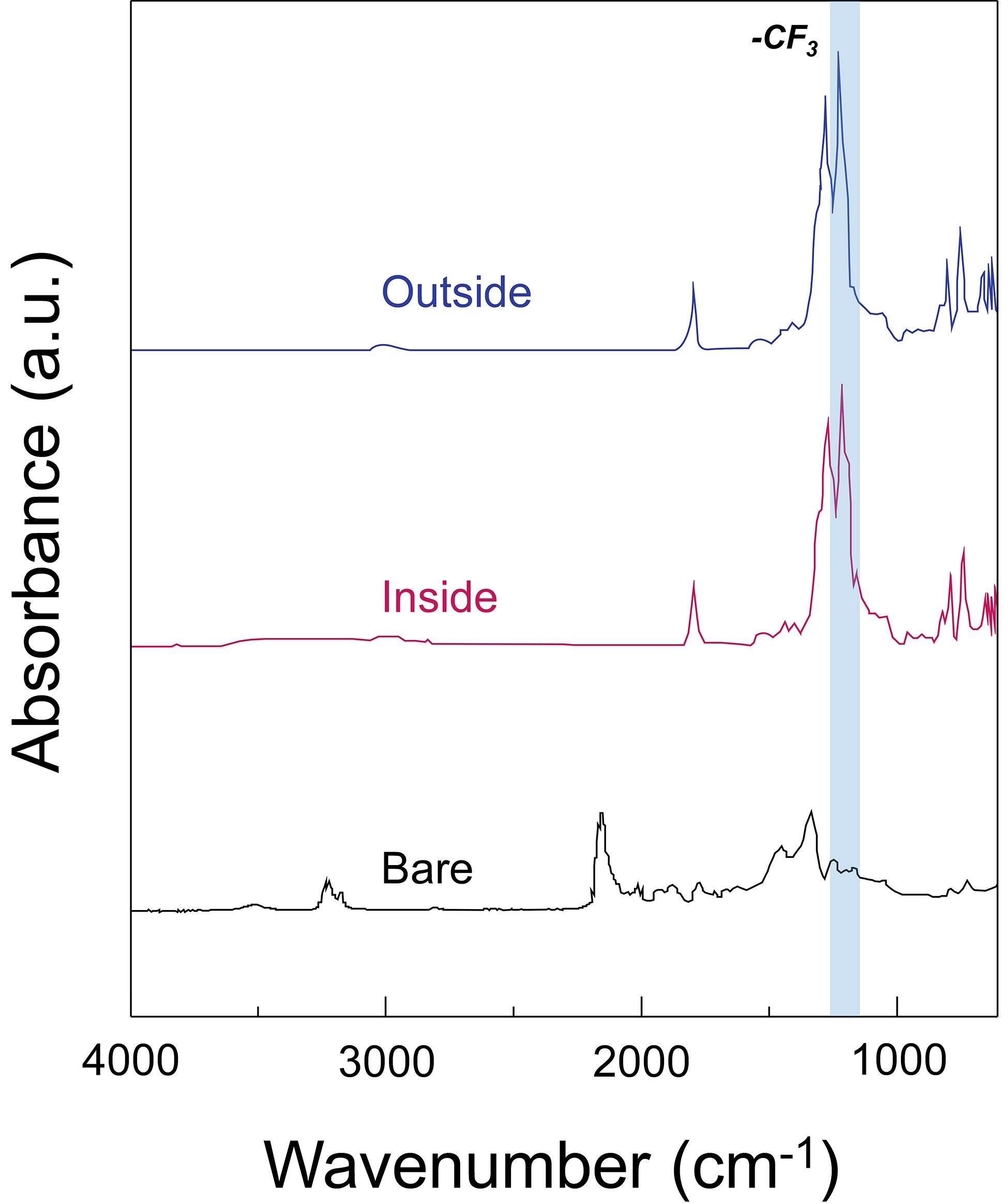


**Figure S2.** FTIR spectrum of bare sponge (black), inside surface of PFDMA coated sponge (red), and outside surface of PFDMA coated sponge (blue). The generation of the peak representing the −CF3 functional group indicates the successful deposition of PFDMA.

**3. Experimental setup for abrasion test**


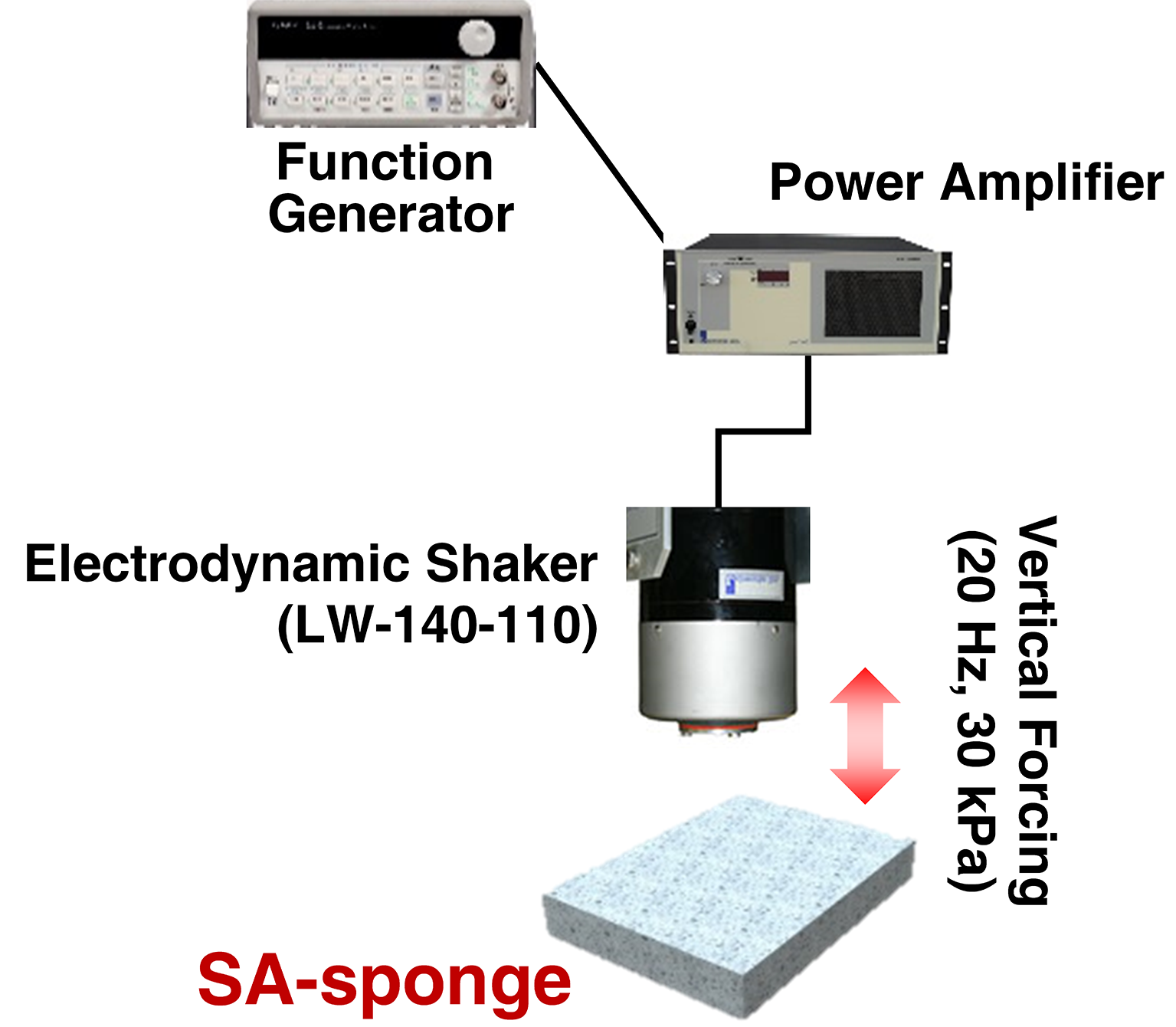


**Figure S3.** Experimental setup for abrasion test. A vibration forcing by electrodynamic shaker (LW-140-110, Labworks Inc., USA) is controlled by function generator and power amplifier.

**4. TEM image for conformal coating by use of the i-CVD**


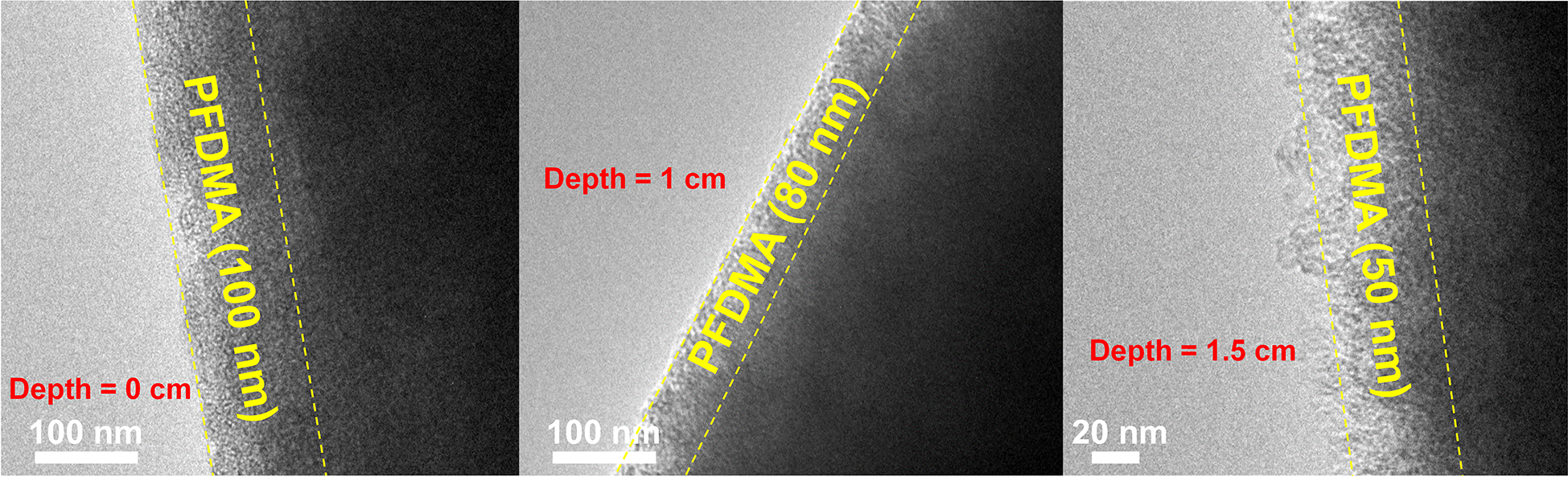


**Figure S4.** TEM image of PFDMA coated surface according to the depth from the bottom.

**5. Thickness of PFDMA thin layer according to depth**


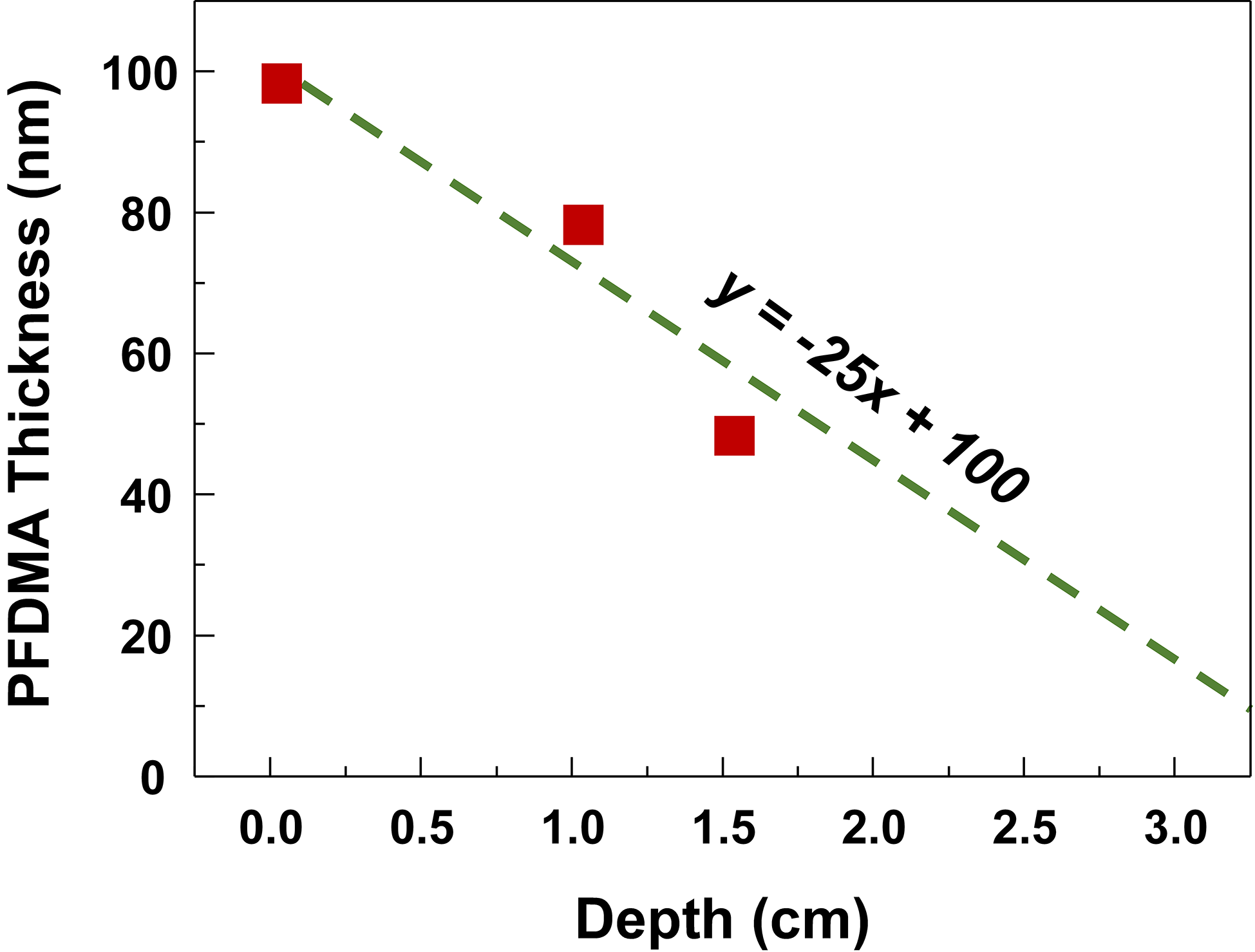


**Figure S5.** Depth profile of the thickness of the PFDMA thin film onto the surface of the sponge.

**6. Video for a superamphiphobic sponge in various liquids**

**Video S1.** Fabricated SA-sponge is submerged in various liquid (olive oil, hexadecane, engine oil, water).
